# Integrated analysis of ceRNA network in hepatocellular carcinoma using bioinformatics analysis

**DOI:** 10.1097/MD.0000000000026194

**Published:** 2021-06-04

**Authors:** Yu Luo, Hongjuan Li, Hongli Huang, Lian Xue, Haiwen Li, Li Liu, Haiyan Fu

**Affiliations:** a Department of Oncology; b Department of Liver Disease, The 3rd People's Hospital of Kunming, Kunming, Yunnan, China.

**Keywords:** biological markers, ceRNA, hepatocellular carcinoma, prognosis

## Abstract

**Background:**

Long noncoding RNAs (lncRNAs) can work as microRNA (miRNA) sponges through a competitive endogenous RNA (ceRNA) mechanism. LncRNAs and miRNAs are important components of competitive endogenous binding, and their expression imbalance in hepatocellular carcinoma (HCC) is closely related to tumor development, diagnosis, and prognosis. This study explored the potential impact of the ceRNA regulatory network in HCC on the prognosis of HCC patients.

**Methods:**

We thoroughly researched the differential expression profiles of lncRNAs, miRNAs, and mRNAs from 2 HCC Gene Expression Omnibus datasets (*GSE98269* and *GSE60502*). Then, a dysregulated ceRNA network was constructed by bioinformatics. In addition, hub genes in the ceRNA network were screened by Cytoscape, these hub genes functional analysis was performed by gene set enrichment analysis, and the expression of these hub genes in tumors and their correlation with patient prognosis were verified with Gene Expression Profiling Interactive Analysis.

**Results:**

A ceRNA network was successfully constructed in this study including 4 differentially expressed (DE) lncRNAs, 7 DEmiRNAs, and 166 DEmRNAs. Importantly, 4 core genes (*CCNA2*, *CHEK1*, *FOXM1*, and *MCM2*) that were significantly associated with HCC prognosis were identified.

**Conclusions:**

Our study provides comprehensive and meaningful insights into HCC tumorigenesis and the underlying molecular mechanisms of ceRNA. Furthermore, the specific ceRNAs can be further used as potential therapeutic targets and prognostic biomarkers for HCC.

## 1 Introduction

The most common malignant tumor of the liver is the metastasis or spread of tumors from other parts of the body.^[1]^ Hepatocellular carcinoma (HCC) is the most common primary liver cancer in liver tissue-derived tumors. The US surveillance, epidemiology, and results database program indicated that HCC holds proportion for 65% of all cases of liver cancers.^[2]^ Men are generally more vulnerable than women, and the disease most commonly occurs in individuals between 30 and 50 years old. HCC causes 662,000 deaths worldwide each year, approximately half of which are in China.^[3,4]^ Therefore, it is necessary to find more biomarkers and therapeutic targets for the treatment of HCC. This study investigated the role of HCC-associated long noncoding RNAs (lncRNAs) work as competitive endogenous RNAs (ceRNAs) in regulating target genes and their effects on the pathogenesis and prognosis of HCC.

Noncoding RNA (ncRNA) is a type of RNA molecule that is not translated into a protein but is omnipresent in organism.^[5]^ LncRNA is a subtype of ncRNA longer than 200 nucleotides and was once thought to be transcriptional noise. Several studies have revealed that lncRNAs play a pivotal role in tumor-related proliferation, invasion, and metastasis.^[6–8]^ Salmena et al proposed a ceRNA hypothesis to reveal the different biological functions of lncRNAs in malignant tumors.^[9]^ A complex post-transcriptional regulatory network was described in which lncRNAs and mRNAs compete with microRNAs (miRNAs) via serving as natural miRNA sponges. A growing number of studies have verified that the lncRNA–miRNA–mRNA regulatory network plays a critical role in the progression and pathogenesis of several tumors, including breast cancer,^[10]^ gallbladder cancer,^[11]^ liver cancer,^[10,12]^ and other malignant tumors.^[13]^ He et al demonstrated that lncRNA FENDRR and lncRNA HAND2-AS1 were hub nodes in a ceRNA network for predicting liver cancer prognosis.^[12]^ Long et al confirmed that lncRNA00205 modulates the expression of EPHX1 through acting as a ceRNA to inhibit miR-184 in hepatocellular carcinoma.^[10]^ Therefore, ceRNAs have a variety of biological functions and it is worth further exploration of its molecular mechanism.

Over the past decade, microarray technology and comprehensive sequencing efforts have revealed the genomic landscapes of common forms of human cancer.^[14]^ Gene Expression Omnibus (*GEO*) is the largest and most comprehensive public gene expression database resource available today. In this study, 2 datasets (*GSE98269*, *GSE60502*) were downloaded from GEO for analysis with the R tool.^[15]^ The screening of differentially expressed genes (*DEGs*), Gene Ontology terms, and gene set enrichment analysis (*GSEA*) were analyzed with the R package. The gene expression profiling interactive analysis (*GEPIA*)^[16]^ database was used to observe changes in the expression levels of these genes between cancer patients and normal people, and the correlations among genes, survival, and disease were confirmed.

## 2 Methods

### 2.1 Sample collection

In this study, 2 datasets (GSE98269, GSE60502) were downloaded from GEO for analysis with the R tool (a GEOquery package).^[15]^ GSE98269 contains miRNA and lncRNA expression data. Using an Agilent microarray, lncRNAs, and miRNAs in HCC and normal liver tissues were profiled. Six samples were selected for lncRNA analysis (3 HCC, 3 normal), and miRNA analysis was performed in 6 samples (3 HCC, 3 normal). From GSE60502, frozen HCC and adjacent nontumorous liver tissues were used for a gene expression profiling study. Affymetrix *U133A* gene chips were used for gene expression profiling. The 18 paired HCC and nontumorous liver tissues were used to determine highly DEGs.

### 2.2 Identification of DEGs

R tool was used in this study. All DEGs were analyzed with the Linear Models for Microarray package^[17]^ and annotated by converting different probe IDs to gene IDs. The ggplot2^[18]^ and pheatmap packages (https://cran.r-project.org/web/packages/pheatmap/index.html) were also used for this analysis. Because the lncRNA is upstream of the ceRNA regulatory network, we have more stringent control over the quality of the lncRNA. For *lncRNAs*, genes significantly dysregulated in HCC compared to matched normal samples were defined by a *P* value < .05 and log2 fold change of >2 (upregulated lncRNAs) or <−2 (downregulated lncRNAs). For *miRNAs* and *mRNAs*, genes significantly dysregulated in HCC compared to matched normal samples were defined by a *P* value <.05 and log2 fold change of > 1 (upregulated genes) or <−1 (downregulated genes).

### 2.3 Construction of the ceRNA network

To verify the function of lncRNAs, miRNAs, and mrnamRNAs in the ceRNA network and further improve the reliability of the ceRNA network, we screened out differential expression lncRNAs (DElncRNAs), differential expression miRNAs (DEmiRNAs), and differential expression mRNAs (DEmRNAs) to construct a co-expression network. Using the miRNet database (https://www.mirnet.ca/), we confirmed the interactions between DElncRNAs and DEmiRNAs. miRNet, which is an integrated platform linking miRNAs, targets, and functions,^[19,20]^ was used to analyze data collected from 4 well-annotated databases, including miRTarBase, TarBase, and miRecords. Then, we predicted the target mRNAs of the differentially expressed miRNAs using miRWalk 3.0 (http://mirwalk.umm.uni-heidelberg.de/). This tool hosts possible binding site interaction information between genes (encompassing the complete sequence) and *miRNAs* resulting from the TarPmiR algorithm.^[21]^ Finally, we identified the differentially targeted genes in our dataset, constructed a ceRNA network using Cytoscape software (version 3.7.0),^[22]^ and analyzed their biological functions.

### 2.4 Functional enrichment analysis

Metascape (http://metascape.org) is an online program designed to develop a set of reliable, productive, and intuitive tools that help the biomedical research community analyze gene/protein lists and make better data-driven decisions.^[23]^ In the present work, metascape was used for enrichment analysis of the functions. Enriched biological processes, cellular components, molecular functions, and Kyoto Encyclopedia of Gene and Genomes (*KEGG*) pathways were used to analyze the target DEGs of the ceRNA network. *P* value <.05 was set as the threshold.

### 2.5 Correlation between hub genes and HCC

We created a protein–protein interaction (PPI) network with STRING (https://string-db.org/).^[24]^ PPI pairs with default parameters were used to construct the PPI network. Hub genes from the PPI network were screened by Cytoscape software, and the most important module was selected by the MCODE plugin with the default parameters (degree cutoff = 2, node score cutoff = 0.2, K – core = 2, and max depth = 100).^[25]^ Besides, we visualized the regulatory relationship between genes and calculated the network topology properties through the degree algorithm in the CytoHubba application.^[26]^ We defined the top 20 genes in the PPI network as hub genes.

### 2.6 Gene set enrichment analysis

GSEA is a calculation method that determines whether an a priori defined set of genes shows statistically significant, concordant differences between 2 biological states.^[27]^ In this work, clusterProfile packages were used for a GSEA of the pathway. The enrichplot,^[28]^ plyr, ggrepel, ggplot2,^[18]^ RColorBrewer, and gridExtra packages were also used for visualizing this analysis. *P* value <.05 was recognized as significantly enriched.

### 2.7 Survival analysis of key genes

GEPIA (http://gepia.cancer-pku.cn/index.html) is an interactive web server used to analyze the TCGA dataset, which includes RNA-seq expression data from 9736 tumor samples and 8587 normal samples, and the Genotype-Tissue Expression projects.^[29]^ In the current study, the hub genes were analyzed to determine their association with prognosis with GEPIA and the TCGA datasets. The hub genes that were significantly associated with HCC patient prognosis were identified (*P* < .01), and the expression levels of these core genes in HCC and normal samples were explored with boxplots.

## 3 Results

### 3.1 Significant expression changes in RNAs in HCC

**Figure 1:**
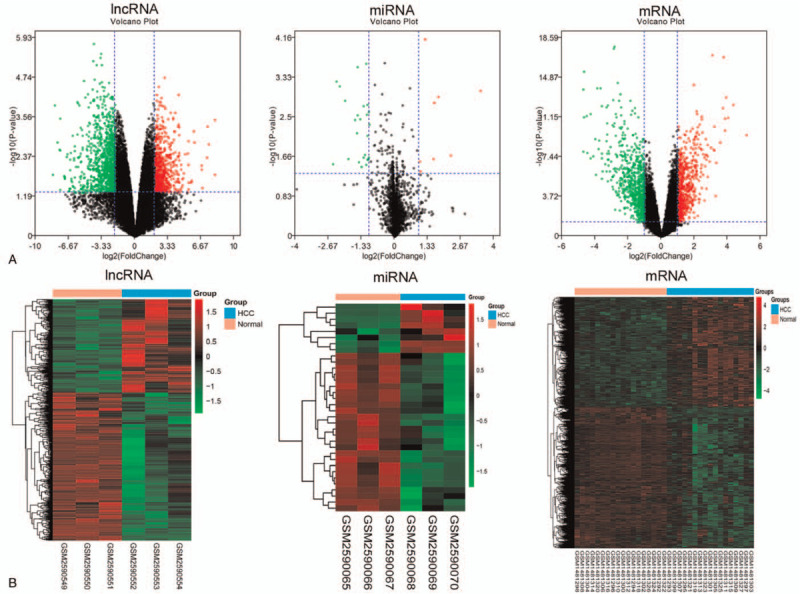
Identification of significant expression changes in RNAs in HCC. A Volcano plot for the DElncRNAs and DEmiRNAs in GSE98269 and DEmRNAs in GSE60502. The black dots represent genes that are not differentially expressed between the HCC samples and normal samples, and the green dots and red dots represent the downregulated and upregulated genes, respectively, in HCC samples. B Heatmap of the DElncRNAs and DEmiRNAs in GSE98269 and DEmRNAs in GSE60502. Genes expressed at high levels are shown in red, and genes expressed at low levels are shown in green.

To determine the RNA levels in HCC, we evaluated the lncRNAs and miRNAs in GSE98269 and mRNAs in GSE60502 and matched normal controls. We analyzed differentially expressed lncRNAs with a cutoff log2 fold change >2 or <−2 and *P* value <.05 and miRNAs and mRNAs with a cutoff log2 fold change >1 or <−1 and *P* value <.05. In contrast to those in the normal control group, 614 lncRNAs were significantly upregulated, and 976 were downregulated; 8 miRNAs were upregulated, and 26 miRNAs were downregulated in GSE98269. In addition, 538 mRNAs were upregulated, and 699 were downregulated in GSE60502. The detailed miRNA differences are documented in Table S1, Supplemental Digital Content. Furthermore, DEncRNAs and mRNAs in the samples are shown using volcano plots (Fig. 1A) and heatmaps (Fig. 1B).

### 3.2 The construction of ceRNA networks

**Figure 2:**
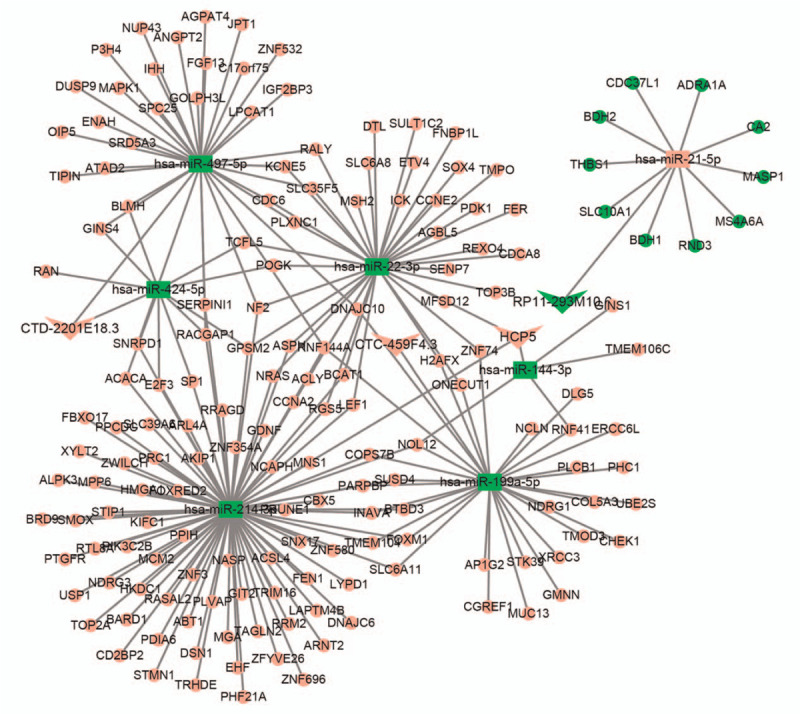
ceRNA network. The triangle represents lncRNAs, the square represents miRNAs, and the circle represents mRNAs. Red nodes represent upregulated genes, and green nodes represent downregulated genes.

The ceRNA networks in this study contain lncRNAs (up)-miRNAs (down)-mRNAs (up) and lncRNAs (down)-miRNAs (up)-mRNAs (down). The DEGs were divided into upregulated RNAs and downregulated RNAs. Then, the miRNet database was used to predict the interactions of microRNAs with lncRNAs. Next, the miRWalk database was used to identify the interactions of microRNAs with mRNAs, and a ceRNA network was constructed with microRNA as the intermediate point. As shown in Figure 2, we successfully constructed 2 ceRNA networks, 1 containing 1 downregulated lncRNA (RP11–293M10.6), 1 upregulated miRNA (hsa-miR-21-5p) and 10 downregulated mRNAs. The other ceRNA network contains 3 upregulated lncRNAs (CTC-459F4.3, CTD-2201E18.3, and HCP5), 6 downregulated microRNAs (hsa-miR-199a-5p, hsa-miR-424-5p, hsa-miR-497-5p, hsa-miR-144-3p, hsa-miR-214-3p, and hsa-miR-22-3p) and 156 upregulated genes. The detailed miRNA differences are documented in Table S2, Supplemental Digital Content.

### 3.3 Functional enrichment analysis and PPI network

**Figure 3:**
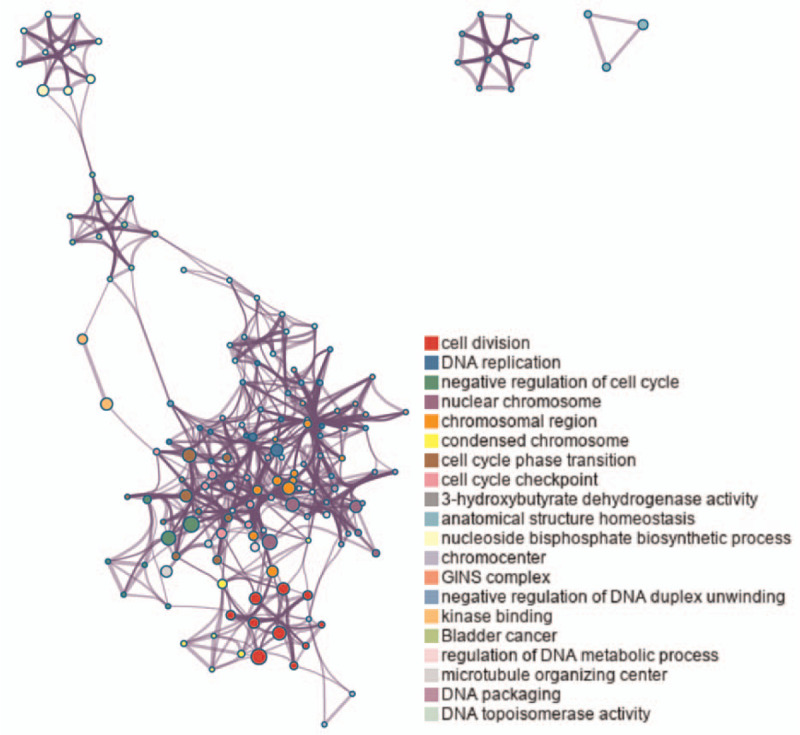
Top 20 clusters with their representative enriched terms (colored by cluster ID), where nodes that share the same cluster ID are typically close to each other.

Metascape was used to analyze the downstream target DEGs of the ceRNA network. For a given gene list, the Gene Ontology enrichment analysis demonstrated that the upregulated DEGs were dramatically concentrated in cell division, DNA repair, and nuclear chromosomes. The downregulated DEGs were mainly enriched in the ketone body biosynthetic process, cellular ketone body metabolic process, and positive regulation of synaptic transmission, GABAergic. KEGG analysis revealed that the upregulated DEGs were dramatically concentrated in the cell cycle, central carbon metabolism in cancer, and pathways in cancer. The downregulated DEGs were enriched in the synthesis and degradation of ketone bodies, butanoate metabolism, and bile secretion (Table 1 and Fig. 3).

**Table 1:** Results of the top 10 GO terms and the top 10 KEGG pathway analyses.

Category	Term	Description	Gene counts	LogP
Upregulated *DEGs*				
GO: 0051301	BP	Cell division	23	−11
GO: 0007059	BP	Chromosome segregation	17	−10
GO: 0006260	BP	DNA replication	15	−9.4
GO: 0000228	CC	Nuclear chromosome	19	−7.8
GO: 0098813	BP	Nuclear chromosome segregation	13	−7.7
GO: 0045786	BP	Negative regulation of cell cycle	19	−7.4
GO: 0000819	BP	Sister chromatid segregation	11	−7.3
GO: 0098687	CC	Chromosomal region	14	−7.2
GO: 0000280	BP	Nuclear division	15	−7.1
GO: 0044454	CC	Nuclear chromosome part	17	−6.7
hsa04110	*KEGG*	Cell cycle	6	−3.8
hsa05215	*KEGG*	Prostate cancer	5	−3.6
hsa05224	*KEGG*	Breast cancer	6	−3.5
hsa05230	*KEGG*	Central carbon metabolism in cancer	4	−3.1
hsa05216	*KEGG*	Thyroid cancer	3	−3.1
hsa05218	*KEGG*	Melanoma	4	−3
hsa05200	*KEGG*	Pathways in cancer	9	−3
hsa05219	*KEGG*	Bladder cancer	3	−2.6
hsa03440	*KEGG*	Homologous recombination	3	−2.6
hsa05161	*KEGG*	Hepatitis B	5	−2.6
Downregulated *DEGs*				
GO: 0046951	BP	Ketone body biosynthetic process	2	−5.3
GO: 0046950	BP	Cellular ketone body metabolic process	2	−5.1
GO: 0032230	BP	Positive regulation of synaptic transmission, GABAergic	2	−5
GO: 1902224	BP	Ketone body metabolic process	2	−5
GO: 0032228	BP	Regulation of synaptic transmission, GABAergic	2	−4.1
GO: 0051932	BP	Synaptic transmission, GABAergic	2	−3.8
GO: 0099617	CC	Matrix side of mitochondrial inner membrane	1	−3.4
GO: 0001985	BP	Negative regulation of heart rate involved in baroreceptor response to increased systemic arterial b	1	−3.4
GO: 0001983	BP	Baroreceptor response to increased systemic arterial blood pressure	1	−3.4
GO: 0001978	BP	regulation of systemic arterial blood Pressure by carotid sinus baroreceptor feedback	1	−3.4
hsa00072	*KEGG*	Synthesis and degradation of ketone bodies	2	−5.2
hsa00650	*KEGG*	Butanoate metabolism	2	−4.2
hsa04976	*KEGG*	Bile secretion	2	−3.4
hsa00910	*KEGG*	Nitrogen metabolism	1	−2.2
hsa04964	*KEGG*	Proximal tubule bicarbonate reclamation	1	−2
hsa04966	*KEGG*	Collecting duct acid secretion	1	−2
hsa05219	*KEGG*	Bladder cancer	1	−1.8
hsa05144	*KEGG*	Malaria	1	−1.7
hsa05150	*KEGG*	Staphylococcus aureus infection	1	−1.6
hsa04115	*KEGG*	p53 signaling pathway	1	−1.5

**Figure 4:**
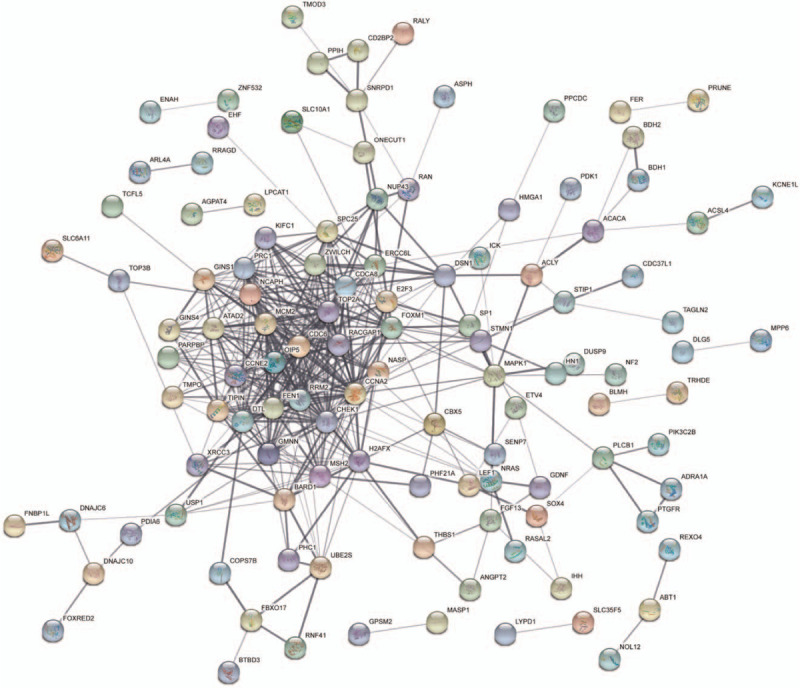
PPI network of *DEGs*.

**Figure 5:**
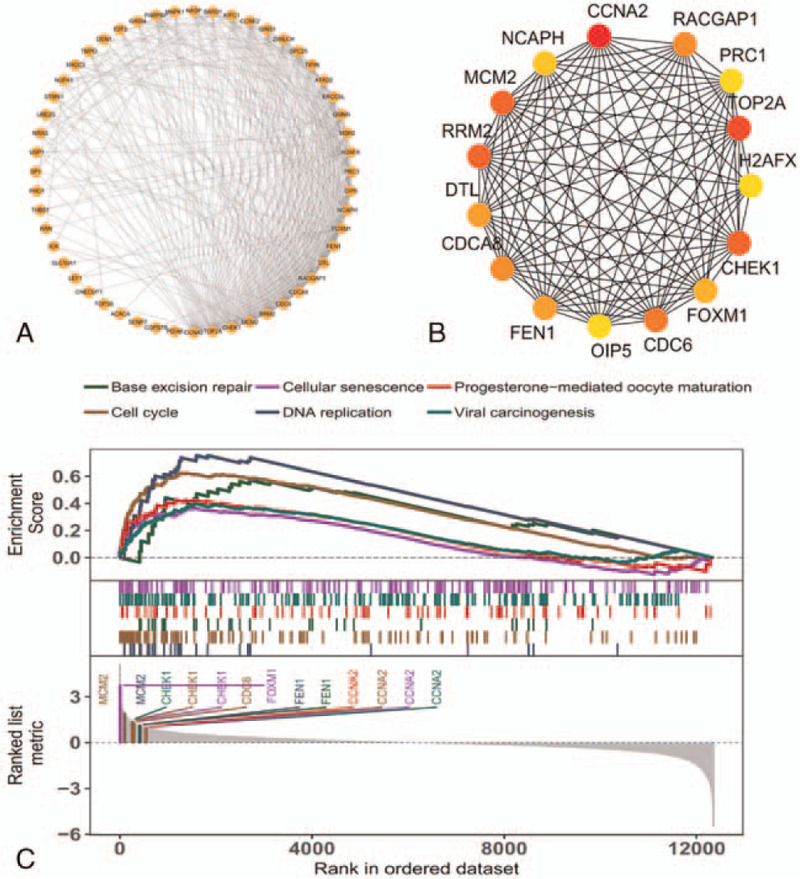
Hub gene analysis and *GSEA*. A The Highest connectivity modules according to MCODE analysis. B Top 15 hub genes according to CytoHubba. C *GSEA* of hub genes.

For analyzing the PPI network, the files from the STRING database were imported into Cytoscape software with 114 nodes and 443 edges mapped (Fig. 4). We used the MCODE plugin to analyze the highest connectivity with 52 nodes and 367 edges of the self-network, as shown in Figure 5A. In addition, 15 hub genes were determined by the CytoHubba plugin using the degree method and included *CCNA2*, *TOP2A*, *RRM2*, *MCM2*, *CHEK1*, *CDC6*, *RACGAP1*, *CDCA8*, *FEN1*, *DTL*, *FOXM1*, *NCAPH*, *PRC1*, *H2AFX*, and *OIP5* (Fig. 5B). The hub genes were also found in the module. In our data, all of the hub genes were upregulated.

To further understand the biological functions of these hub genes, *GSEA* was performed. We enriched 95 KEGG signaling pathways by GSEA. Then, we presented the signal pathways of 8 selected genes. As shown in Figure 5C, the 6 hub genes (*CCNA2*, *MCM2*, *CHEK1*, *CDC6*, *FEN1*, and *FOXM1*) are enriched in the related signal pathways, including cell cycle, DNA repair, and cellular senescence. Due to the importance of these genes and the changes in these signaling pathways, we believe that they are involved in the occurrence and development of HCC.

### 3.4 The DEG alterations identified may play an important role in HCC patient survival

**Figure 6:**
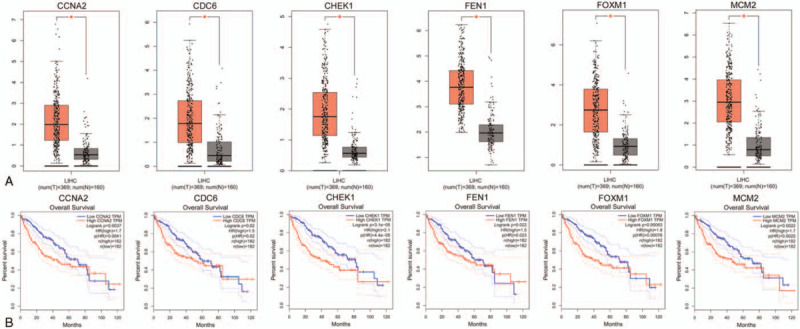
Prognostic values and expression levels of the 4 hub genes in HCC patients. A Expression level of 6 hub genes in HCC and normal tissues. B Prognostic value of 6 hub genes in HCC.

To further confirm the relationship between these 6 hub genes and the prognosis of HCC patients, a survival curve and expression levels of the hub genes were detected by GEPIA. As shown in Figure 6a, 6 genes are upregulated in HCC. This is consistent with our chip analysis results.

Survival analysis results suggest that high expression levels of CCNA2 (HR = 1.7, Logrank p = 0.0037), CHEK1 (HR = 2.1, Logrank p = 3.1e−05), FOXM1 (HR = 1.8, Logrank p = 0.00063) and MCM2 (HR = 1.7, Logrank p = 0.0022) are associated with worse overall survival (OS) in HCC patients (*P* < .01) (Fig. 6b).

## 4 Discussion

Currently, HCC has become the second leading cause of cancer death worldwide due to its extremely high mortality rate.^[30]^ Most patients with the asymptomatic form of HCC are diagnosed as terminal. Therefore, the elucidation of the molecular mechanisms of HCC, for the identification of new therapeutic targets, improve the clinical efficacy in patients with HCC is important. It is well known that most ncRNAs play a role by regulating the expression of some important genes. Numerous studies have shown that miRNAs and lncRNAs have complex and closely related regulatory networks and play an important role in the occurrence and development of tumors.^[31,32]^ According to the ceRNA hypothesis, lncRNAs, whose sequences are similar to the target miRNAs, are capable of regulating the expression of mRNAs by serving as miRNA sponges.^[9]^ There have been some reports about the comprehensive expression profiles and related ceRNA networks in the process of tumorigenesis or differentiation, for example, in bladder cancer,^[33,34]^ glioblastoma,^[35,36]^ and esophageal cancer.^[37,38]^

However, few studies have focused on ceRNAs predict the prognosis of HCC,^[36–38]^ such as Bai et al used the ceRNA network to reveal the potential prognostic cytoplasmic lncRNA involved in HCC progression.^[36]^ In addition, lncRNAs, miRNAs, and mRNAs associated with HCC can be treated as molecular biomarkers. Based on this background information and hypothesis, we systematically analyzed the complex interactions of interrelated mRNAs, miRNAs, and lncRNAs to provide a network for revealing deregulated lncRNAs. We analyzed Agilent and Affymetrix gene chip data and identified differential expression profiles of lncRNAs, microRNAs, and mRNAs in patients with HCC and normal controls and further constructed a ceRNA network. In our ceRNA work, we found 4 lncRNAs, 7 miRNAs, and 166 mRNAs with significant expression changes. Functional enrichment analysis showed that the genes were mainly enriched in cell division, DNA repair, and pathways in cancer. To further analyze the key genes related to HCC, we constructed a PPI network and performed GSEA. More significantly, the results suggested that CCNA2, CHEK1, FOXM1, and MCM2, which are closely related to the OS of HCC patients, are core genes. The results suggest that at the transcriptional level, a specific group of miRNAs and lncRNAs may be involved in the regulatory mechanism of HCC development.

In our ceRNA network, lncRNA HCP5 had the highest connection among the DElncRNAs (lncRNA CTC-459F4.3, lncRNA CTD-2201E18.3, and lncRNA RP11-293M10.6). Therefore, we hypothesize that it may play an important role in the development and prognosis of HCC. lncRNA HCP5 is involved in the ceRNA regulatory network of various tumors. Wei et al showed that lncRNA HCP5 overexpression inhibited the development of skin melanoma through a ceRNA mechanism.^[39]^ Jiang et al reported that lncRNA HCP5 was highly expressed and promoted lung adenocarcinoma metastasis via the has-miR-203/SNAI axis.^[40]^ Yu et al suggested that lncRNA HCP5 was overexpressed in cervical cancer tissues and promoted the development of cervical cancer by regulating MACC1 via suppressing microRNA-15a.^[41]^ Our study also showed that lncRNA HCP5 expression was 4.7-fold higher in HCC tissues than in paired normal tissues. Moreover, we found that lncRNA HCP5 might compete with 2 core DEmiRNAs (hsa-miR-214-3p and hsa-miR-22-3p) to regulate target DEmRNA expression in HCC.

MiRNA is an indispensable component of cancer development and prognosis. We found that the DEmiRNA hsa-miR-214-3p had the highest connection among the DEmiRNAs in the ceRNA network. This may indicate that hsa-miR-214-3p may be involved in the occurrence and prognosis of HCC. Wang et al. reported that has-miR-214-3p was expressed at low levels and promoted the development of epithelial ovarian cancer.^[42]^ Kitdumrongthum et al demonstrated that hsa-miR-214-3p was downregulated 1000- to 2000-fold in cholangiocarcinoma exosomes.^[43]^ Yang et al showed that has-miR-214-3p deficiency exacerbated cardiac fibrosis.^[44]^ The downregulation of miR-214-3p expression inhibited the proliferation and invasion of HCC cells^[45]^ and inhibited the progression of HCC by directly downregulating MELK expression.^[46]^ Consistent with previous studies, our study indicated that miR-214-3p is downregulated by 2-fold in HCC and may affect HCC prognosis by directly regulating downstream target genes.

In addition, we found that the downregulated DEmiRNA hsa-miR-199a-5p, hsa-miR-424-5p, and hsa-miR-497-5p also has related reports. Jiang et al. found that hsa-miR-199a-5p might be biomarker in the occurrence of HCC.^[47]^ In hepatitis B virus-associated hepatocellular carcinoma (HCC), the low-level expression of hsa-miR-199a-5p lead to the high-level expression of CDK4 and IGF2 mRNA.^[48]^ Wang et al indicated that hsa-miR-424-5p with high accurate prediction.^[49]^ CHEK1-hsa-mir-497-5p was identified as significant interactions, and hsa-mir-497-5p downregulated in hepatocellular carcinoma and display favorable prognostic roles.^[50]^ Besides, there are many miRNAs were considered to be biomarker in HCC.^[51–53]^

For prognostic DEmRNAs, CCNA2 had the highest connection among the prognostic DEmRNAs in the ceRNA network. CCNA2 is a cyclin that controls both the G1/S and G2/M transition phases of the cell cycle.^[54]^ This is consistent with the results of our GSEA, which indicates that CCNA2 is significantly enriched during the cell cycle. Gao et al showed that high CCNA2 expression was a prognostic biomarker for ER+ breast cancer.^[55]^ Gan reported that CCNA2 was an oncogenic gene that was significantly overexpressed in colorectal cancer and regulated cancer cell growth and apoptosis.^[56]^ CCNA2 has also been associated with poor prognosis in HCC.^[57]^ Argininosuccinate lyase interacts with CCNA2 in the cytoplasm and modulates the growth of liver tumor cells.^[58]^ Consistent with previous studies, our study indicated that CCNA2 is upregulated by 2-fold in HCC, and the survival analysis demonstrated that HCC exhibiting high CCNA2 expression levels presented a poor prognosis (HR = 1.7, Logrank p = 0.0037).

Although our ceRNAs network can recognize many HCC-related lncRNAs, miRNAs, and mRNAs, it is still limited. First, the microarray data for HCC expression in the public GEO dataset are insufficient. The results of this study were obtained mainly through a comprehensive analysis of the GEO database, and the consistency of the sample types is only guaranteed as far as possible. Second, it should be noted that the regulatory networks or mechanisms analyzed in this study were only bioinformatics predictions. However, we believe that the genes in the current ceRNAs network are important in HCC. Therefore, our results need to be further verified in vivo, in vitro experiments, and clinical practice, which is the main focus of our team in the future.

## 5 Conclusions

In conclusion, based on a comprehensive analysis of the HCC GEO dataset, we successfully constructed a ceRNA network including 4 DElncRNAs, 7 DEmiRNAs, and 166 DEmRNAs. Importantly, 4 core genes (CCNA2, CHEK1, FOXM1, and MCM2) that were significantly associated with HCC prognosis were identified. Our study provides comprehensive and meaningful insights into HCC tumorigenesis and the underlying molecular mechanisms of ceRNAs. In addition, the candidate ceRNAs can serve as potential therapeutic targets and prognostic biomarkers for HCC.

## Acknowledgments

We acknowledge Dr Qinggang Hao, who helped to improve the scientific quality of this study.

## Author contributions

**Conceptualization:** Haiyan Fu.

**Data curation:** Yu Luo, Hongjuan Li, Lian Xue, Haiyan Fu.

**Formal analysis:** Yu Luo, Hongjuan Li, Haiyan Fu.

**Funding acquisition:** Haiyan Fu.

**Software:** Hongli Huang, Haiwen Li, Li Liu.

**Writing – original draft:** Yu Luo, Hongjuan Li, Hongli Huang, Lian Xue, Haiwen Li, Li Liu, Haiyan Fu.

**Writing – review & editing:** Yu Luo, Hongjuan Li, Hongli Huang, Lian Xue, Haiwen Li, Li Liu, Haiyan Fu.

## Supplementary Material

Supplemental Digital Content

## Supplementary Material

Supplemental Digital Content
